# Transcribing Genes the Hard Way: *In Vitro* Reconstitution of Nanoarchaeal RNA Polymerase Reveals Unusual Active Site Properties

**DOI:** 10.3389/fmolb.2021.669314

**Published:** 2021-05-11

**Authors:** Sven Nottebaum, Robert O. J. Weinzierl

**Affiliations:** ^1^ Department of Life Sciences, Imperial College London, London, United Kingdom; ^2^ Orthomol Pharmazeutische Vertriebs GmbH, Langenfeld, Germany

**Keywords:** archaea, nanoarchaea, RNA polymerase, catalytic center, active site, high-throughput assay, sparse matrix sampling, fluoride

## Abstract

Nanoarchaea represent a highly diverged archaeal phylum that displays many unusual biological features. The *Nanoarchaeum equitans* genome encodes a complete set of RNA polymerase (RNAP) subunits and basal factors. Several of the standard motifs in the active center contain radical substitutions that are normally expected to render the polymerase catalytically inactive. Here we show that, despite these unusual features, a RNAP reconstituted from recombinant *Nanoarchaeum* subunits is transcriptionally active. Using a sparse-matrix high-throughput screening method we identified an atypical stringent requirement for fluoride ions to maximize its activity under *in vitro* transcription conditions.

## Introduction

The basal transcriptional machineries of Archaea are intriguingly similar to the core components of the eukaryotic RNA polymerase II (RNAPII) transcriptional machinery (Cramer et al., 2001). This close similarity to eukaryotic systems, combined with the greater experimental accessibility, has established archaeal systems as key model systems for in-depth structure/function analyses of the transcriptional machinery (Werner and Weinzierl 2002; Ouhammouch et al., 2004; Werner et al., 2006; Naji et al., 2007; Hirata et al., 2008; Tan et al., 2008; Thomm et al., 2009; Weinzierl 2013; Fouqueau et al., 2018; Blombach et al., 2019; Wenck and Santangelo, 2020). Apart from serving as model systems for eukaryotic systems, archaea also include numerous examples of extremophiles that do not fit the general pattern (Adam et al., 2017). Such species often provide unusual examples of molecular organization that have the capacity of enlarging our understanding of fundamental molecular mechanisms by illustrating the degree of flexibility that is possible, or by providing examples for achieving the same goal in a variety of alternative ways (Coker, 2019). Some of the best-known examples include the adaption of enzymes to operate in high-salt environments (halophiles), over a wide range of temperatures (psychrophile, mesophiles, thermophiles, hyperthermophiles), or at low or high pH (acidophiles and alkaliphiles, respectively). Another interesting class of archaea are the evolutionary “outliers”, such as *Methanopyrus kandleri*, *Cenarchaeum symbiosum,* and *Nanoarchaeum equitans*. The phylogenetic classification of these species is contentious, and their protein sequences frequently contain unique and unusual substitutions that are not shared by other archaea. Such unorthodox features raise many, yet unanswered, questions regarding the evolutionary origin of such species (deep-branching evolutionary ancestry or recent degeneracy?) and often challenge fundamental concepts of apparently well-understood enzymatic pathways and mechanisms (Randau et al., 2005; Randau et al., 2008).

Here we focus on the molecular organization and properties of the RNAP from the hyperthermophile *Nanoarchaeum equitans* (from here on abbreviated as *N. equitans*, or “*n*” as a prefix). *N. equitans* is a highly unusual archaeon because of its diminutive size (0.35–0.5 μm cell diameter), drastically reduced genome and parasitic lifestyle (Huber et al., 2002; Waters et al., 2003; Forterre et al., 2009; Rawle et al., 2017). The genome lacks most genes that are required to produce cellular precursors, such as amino acids, nucleotides, cofactors, and lipids. These are most likely imported directly from the host cell, the crenarchaeote *Ignicoccus hospitalis*. Depending on the criteria chosen, *N. equitans* has been plausibly classified as a new and early diverging archaeal phylum (the “Nanoarchaeota” (Huber et al., 2003)), a sister branch of the Crenarchaea (Ciccarelli et al., 2006), or as a fast-evolving Euryarchaeon (Brochier et al., 2005). Recent studies have demonstrated that Nanoarchaea are widespread and occur in a variety of locations, including mesophilic and halophilic environments (Hohn et al., 2002; McCliment et al., 2006; Probst et al., 2017; Tully et al., 2018; Zhou et al., 2020).

**FIGURE 1:**
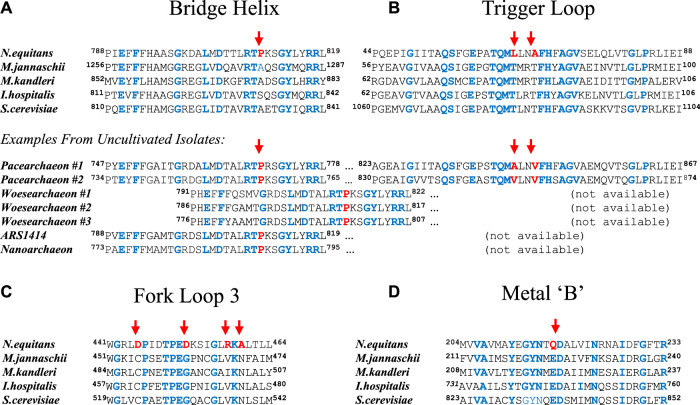
Substitutions in key regions and domains of the nanoarchaeal RNAP catalytic site. Sequences from four archaeal (*Nanoarchaeum equitans*, *Methanocaldococcus jannaschii*, *Methanopyrus kandleri*, and *Ignicoccus hospitalis*) and one eukaryotic (*Saccharomyces cerevisiae*; RNA polymerase II) species are shown in all panels. Unusual substitutions in the nanoarchaeal sequence are highlighted with a red arrow. Residues identical in all sequences shown are highlighted in blue. The beginning and end positions of the sequences shown relative to the full length protein sequence are indicated as superscripts **(A)** Alignment of Bridge Helix sequences. *N. equitans* (SeqID: AAR39345.1), *M. jannaschii* (SeqID: WP_064,496,945.1), *M. kandleri* (SeqID: AAM01900.1), *I. hospitalis* (SeqID WP_011,998,279.1) and *S. cerevisiae* (SeqID: NP_010141.1). Below, additional examples from uncultivated and yet unnamed species are shown (*Candidatus Pacearchaeota archaeon #1* [SeqID: MAG61561.1; RNAP subunit combines A′ and A″ as continuous polypeptide]; *Candidatus Pacearchaeota archaeon #2* [isolate CG_2015–01t_39_43; SeqID: NCO11196.1; RNAP subunit combines A′ and A″ as continuous polypeptide]; *Candidatus Woesearchaeota archaeon* #1 [CG1_02_47_18; SeqID: OIO63522.1; A′ only) (Probst et al., 2017)]; *Candidatus Woesearchaeota archaeon#2* [isolate SpSt-512; SeqID: HGS79070.1; A′ only) (Zhou et al., 2020)]; *Candidatus Woesearchaeota archaeon#3* [isolate SpSt-1178; SeqID: HDP74066.1) (Zhou et al., 2020)]; Archaeal isolate ARS1414 [SeqID: MAG50098.1; A′ only) (Tully et al., 2018)], Nanoarchaeota archaeon [SeqID: NTV23449.1; Breister et al.]) **(B)** Alignment of Trigger Loop sequences. *N. equitans* (SeqID: AAR39272.1), *M. jannaschii* (SeqID: WP_010,870,556.1), *M. kandleri* (SeqID: WP_0,11,019,054.1), *I. hospitalis* (SeqID WP_052,570,437.1), and *S. cerevisiae* (SeqID: NP_010141.1) **(C)** Sequence alignment of the Fork-Loop 3 motif. *N. equitans* (SeqID: AAR39027.1), *M. jannaschii* (SeqID: Q58444.1), *M. kandleri* (SeqID: WP_088,335,828.1), *I. hospitalis* (SeqID: WP_052,570,488.1), and *S. cerevisiae* (SeqID: AAA68096.1) **(D)** Sequence alignment of the Metal-B motif. *N. equitans* (SeqID: AAR39011.1), *M. jannaschii* (SeqID: Q60181.1), *M. kandleri* (SeqID: WP_193,333,232.1), *I. hospitalis* (SeqID: WP_052,570,488.1), and *S. cerevisiae* (SeqID: AAA68096.1).

**FIGURE 2:**
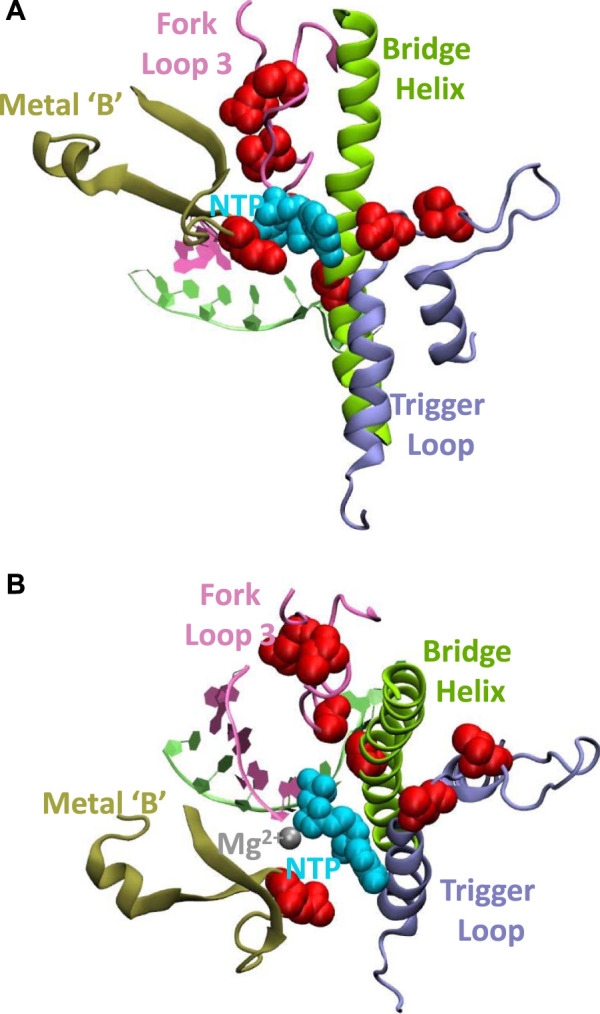
Spatial arrangement of nanoarchaeal-specific substitutions within the catalytic site of RNAP. The Bridge Helix (BH) is shown in green, the Trigger Loop (TL) in blue and Fork Loop 3 (FL-3) in purple. The nucleotide triphosphate is represented as a cyan space-filling model. Positions substituted in *N. equitans* are shown as red space-filling van der Waals representations.

The *N. equitans* genome encodes a full complement of all RNA polymerase (RNAP) subunits and basal factors TBP, TFB, TFE, and TF-S (Huber et al., 2002; Waters et al., 2003). Considering the minimal size of the genome, the presence of a set of genes encoding a complete basal transcriptional machinery strongly suggests that *N. equitans* is fully capable of transcribing its own genome. We observe, however, a distinct set of substitutions in several key positions of the *ne*RNAP catalytic center that are of a unique and radical nature and raise the question whether such an enzyme could display a substantial amount of catalytic activity. The Bridge Helix (BH), Trigger Loop (TL), Fork Loop-3 (FL-3), as well as the “Metal B” binding domain (Me-B; responsible for positioning one of the two catalytically active Mg^2+^ ions) display substitutions in positions that are typically absolutely or highly conserved in all other archaeal and eukaryotic RNAPs (Figure 1). Some of these substitutions (such as the presence of a proline in the Bridge Helix (BH); Figure 1A) are predicted to have highly disruptive, non-local effects by destabilizing the *a*-helical integrity of such a key element in a particularly critical position (Tan et al., 2008; Weinzierl, 2010b, Weinzierl, 2010a, Weinzierl, 2011). Although proline substitutions in particular places of BH results in a substantial increase the specific activity of the structurally closely related euryarchaeal RNAP from *Methanocaldococcus jannaschii* (*mj*RNAP) (Tan et al., 2008; Weinzierl, 2010b), a proline located in the position characteristic for *ne*A′ causes a substantial drop in activity in *mj*RNAP (Tan et al., 2008). Several other unusual substitutions in other key elements of the catalytic site (Cramer et al., 2001) are evident, including the Trigger Loop (TL; Figure 1B), Fork Loop-3 (FL3; Figure 1C) and the Metal-B motif required to coordinate the Mg^2+^ ions facilitating the various types of catalytic chemistries (Sosunov et al., 2003); Me-B; Figure 1D). All these nanoarchaeal substitutions are spatially in close vicinity within the catalytic site of RNAP (Figure 2). Based on our current understanding of the structural basis of the nucleotide addition cycle, such substitutions would be predicted to have a substantially deleterious effect on the catalytic function of the *ne*RNAP active site. In comparison, the RNAP of the archaeon *I. hospitalis* - the host to *N. equitans* - does not encode any of these unusual substitutions found in the *ne*RNAP (Figure 1), thus essentially ruling out that the substitutions are required to survive in a particular environment.

Recent large-scale sequencing efforts have demonstrated that similar unusual substitution patterns can been found in hundreds of sequence samples derived from fresh- and marine water sources (Probst et al., 2017; Tully et al., 2018; Zhou et al., 2020). Several data base entries, labeled as yet unnamed representatives of Woesearchaea or Pacearchaea, show the same types of substitutions as originally found in *N. equitans* (Figure 1A). Although the Bridge Helix and Trigger Loop are usually encoded by separate subunits of archaeal RNAPs (A′ and A″, respectively) - and can therefore usually not be allocated to the same species in environmental sequencing samples - there are two pacearchaeal sequences where A’ and A” appear to be fused into a single subunit (Figure 1A; directly comparable to the eukaryotic large RNAP subunits). We can therefore see from these examples, that - like in *N. equitans* - the specific substitution pattern in both Bridge Helix and Trigger Loop are encoded within an RNAP subunit from the same species. This suggests that, although for a long time considered unusual, *N. equitans* is actually a fairly typical representative of a larger group of archaea (including Pacearchaea, Woesearchaea etc.) that display comparable, but structurally diverged RNAP active site architectures.

The goal of this study was to determine whether the RNAP encoded by the *N. equitans* genome was 1) enzymatically active and 2) to what extent the substitution pattern resulted in altered catalytic properties.

## Materials and Methods

### Identification of neRNAP Subunits and Basal Transcription Factors

*ne*RNAP subunit-encoding open reading frames were identified using existing data base annotations and tblastn searches of the *Nanoarchaeum equitans* genome sequence (SeqID: AE017199.1; see Supplementary Table S1 for more details).

### Markov chain Monte Carlo Simulations

Markov Chain Monte Carlo MCMC simulations were carried out as described previously (Sullivan and Weinzierl, 2020). Briefly, the simulations employed the PROFASI forcefield in the PHAISTOS package (Boomsma et al., 2013). Due to the origin of the proteins from hyperthermophilic organisms, the simulation temperature was set to 355 K (81.85°C). The resulting trajectory data (based on 50,000 calculated states per simulation) was analyzed for secondary structure elements using cpptraj (Roe and Cheatham, 2013) and processed/visualized with custom scripts on *Python* Jupyter notebooks.

### *In Vitro* Reconstitution *ne*RNAP

The protein-coding portions of RNAP subunits A′, A″, B′, B″, D, H, L, N and *p* were PCR amplified from purified *N. equitans* genomic DNA (a gift from Prof. M. Thomm, University of Regensburg) as full-length, non-tagged sequences and cloned as NdeI-BamHI (*ne*A″, *ne*B′, *ne*B″, *ne*F, *ne*K), or NdeI-EcoRI (*ne*A′, *ne*D, *ne*E, *ne*H, *ne*L, *ne*N, *ne*P) fragments into the bacterial expression vector pET21a. Recombinant proteins were expressed with IPTG-induction in *E. coli* BL21-DE3 Rosetta 2 (Merck) under standard conditions (Werner and Weinzierl, 2002). Subunits *ne*A′, *ne*A″, *ne*B’ and *ne*B″ were purified as insoluble inclusion bodies. Briefly, bacterial cells expressing these recombinant subunits were resuspended in T/G_0_ (25 mM Tris-base, 200 mM glycine, 10 mM magnesium acetate, 100 μM zinc acetate, 14 mM *ß*-mercaptoethanol and 10% glycerol at pH7.5) and sonicated. The inclusion bodies were washed extensively with 1 x deoxycholate buffer (1 mg/ml deoxycholate, 15 mM *ß*-mercaptoethanol) and water/15 mM *ß*-mercaptoethanol before solubilizing them in T/G_0_ in the presence of saturating urea or 6 M guanidine-hydrochloride. Subunits *ne*D, *ne*L, *ne*H, *ne*N and *ne*P were expressed similarly as soluble recombinant proteins. Bacterial cells expressing these recombinant subunits were resuspended in P300 Buffer (300 mM potassium acetate, 20 mM Tris-acetate pH 7.9, 10 mM magnesium acetate, 100 μM zinc acetate, 14 mM *ß*-mercaptoethanol and 10% glycerol) and sonicated. The supernatant containing the solubilized proteins were heat-inactivated of at 70°C for 10 min to precipitate the bacterial proteins present in the extract (the hyperthermophilic *ne* subunits remain completely soluble during this treatment).

The urea-solubilized inclusion bodies, or the soluble subunits, were passed over ∼5 ml SP- or Q-Sepharose (Fast flow, Amersham) in chromatography columns. Proteins were eluted in a salt gradient from T/G0 to T/G1000 using a DuoFlow BioLogic FPLC system (BioRad). The purified subunits were assembled by mixing them in the presence of 8 M urea in a dialysis cell (Slide-A-Lyzer 3500MCOW frames [Pierce], or 96-well microdialyser (SpectraPor) on a Theonix robotic platform (Aviso) for high-throughput assembly (Nottebaum et al., 2008; Weinzierl, 2013)), followed by lowering the urea concentration by gradual dilution in the dialysis buffer (Werner and Weinzierl, 2002; Naji et al., 2007; Nottebaum et al., 2008; Weinzierl, 2013). Equimolar amounts of the large subunits were mixed with small subunits, which were in at least four-fold excess to the large subunits, under denaturing conditions. The highest yield of enzymatically active *ne*RNAP (due to increased folding efficiency) was obtained in the presence of 500 mM salt (either sodium chloride, potassium- or sodium acetate) in the refolding buffer (Supplementary Figure S1). The assembly of large complexes was monitored by analytical size exclusion chromatography on Superose-6 and Superose-12 columns (Amersham) at a flow-rate of 0.5 ml/min. When required, soluble protein complexes were concentrated further using centrifugal YM-50 Centricon (Millipore) units according to the manufacturers instructions.

### *In Vitro* Transcription Assay

Refolded RNAPs were assayed for transcriptional activity by measuring incorporation of *a*-^32^P-UTP into RNA. Refolded RNAP was added to 1 x transcription buffer (1 x TB) containing 500 μM ATP, CTP, GTP, 1 μM UTP, 27 nM *a*-^32^P-UTP (6000 Ci/mmol, Amersham), 1.5 μg nuclease-activated calf thymus DNA (Fluka), 120 mM potassium acetate, 10 mM magnesium acetate, 10 mM Tris-acetate pH 7.5 and 10 mM DTT, which was incubated at 37–65°C for 45 min. The final reaction volume was 50 μL. The reactions were stopped by addition of 15% (w/v) trichloroacetic acid followed by 30 min incubation on ice. The precipitate was collected on 96-well GF/F glass fiber filter plates (Whatman), washed twice with excess 10% TCA, once with 95% ethanol, and quantitated in a scintillation counter in presence of scintillant fluid (Opti-fluor, Packard Bioscience). These steps were fully automated on a Theonyx liquid handling platform (Aviso) (Nottebaum et al., 2008; Weinzierl, 2013). Independent repetitions (“biological replicates”) of the same transcription reaction are reproducible within a 12% error margin. Transcripts originating solely from abortive initiation are not precipitated using this method. Therefore, only transcripts from elongation-competent RNAPs (longer than ∼20 nucleotides) give rise to a signal in this assay.

### High-Throughput “Sparse-Matrix” Sampling

Crystallization buffer sets ICL-1, -3, -4, and -5 (Hampton Research, Aliso Viejo, United States; Supplementary Figures S2A–F) were used as 10 x concentrates for high-throughput transcription assays based on nicked DNA templates as described previously (Werner and Weinzierl, 2002; Nottebaum et al., 2008; Tan et al., 2008; Boomsma et al., 2013). Briefly, the assay measures the incorporation of *a*-^32^P-UTP into acid-insoluble RNA by liquid scintillation counting in a robotically implemented workflow. Similar to the strategy used when employing such buffer sets for crystallization screens, the initial screen was only carried out with one assay per buffer set. Buffer sets that gave high levels of activity were subsequently tested in triplicate to confirm the result.

## Results

### Computational Simulations

**FIGURE 3:**
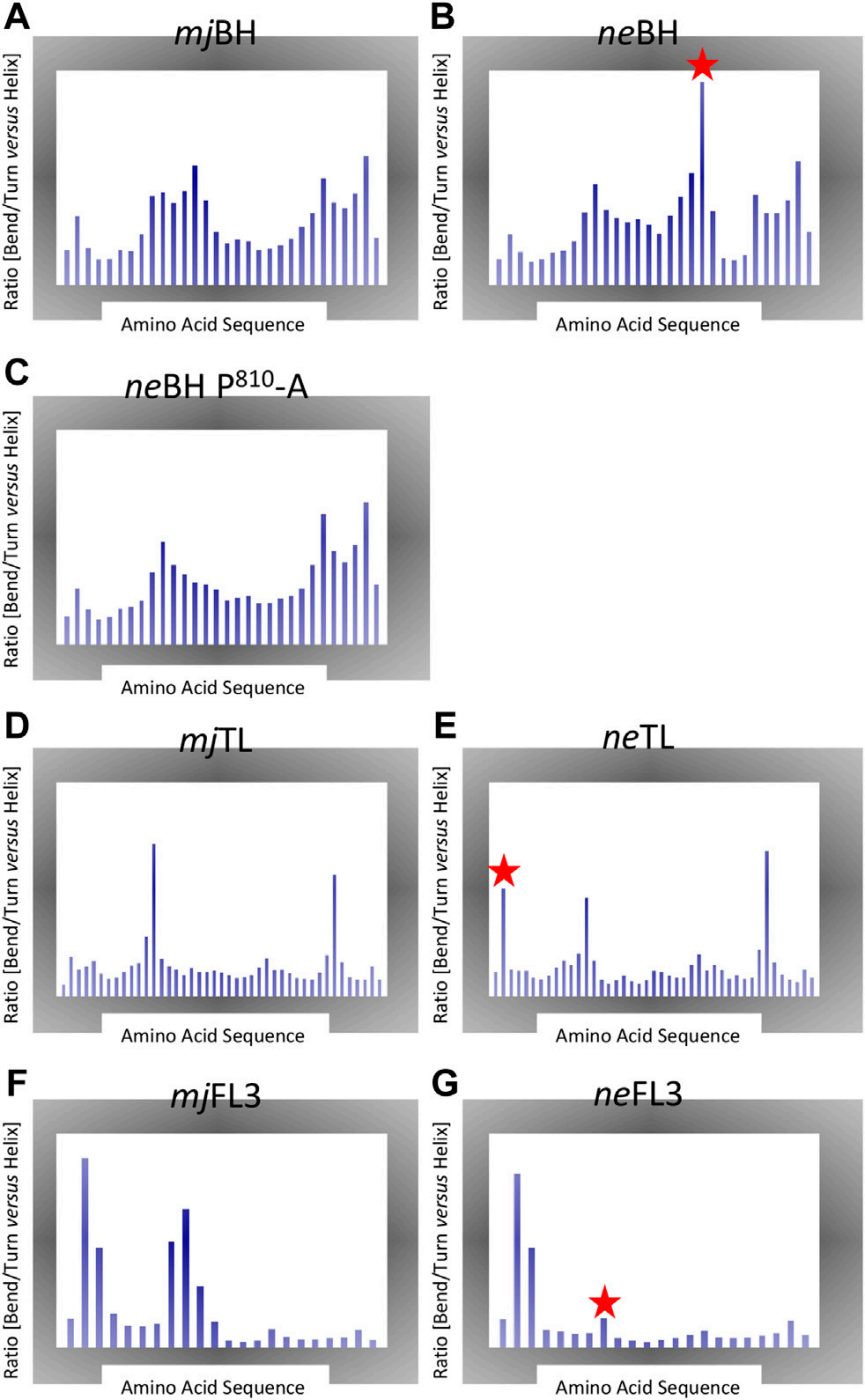
Markov Chain Monte Carlo (MCMC). Each plot shows the ratio of percentage of disordered secondary structure elements (bends, turns) vs. percentage of helical structures (including *a*-, 3–10 and *p* helix data) created during the simulations. Large peaks therefore highlight regions displaying local structural instability. The *x*-axis shows the amino acid positions for each element. Note that the range of *y*-axes is variable between different plot groups. Significant local structural variations present in the *ne* motifs are highlighted with a red star **(A)** Bridge Helix from *M. jannaschii* **(B)** Bridge Helix from *N. equitans* **(C)** Bridge Helix from *M. jannaschii* simulated with *in silico* mutated *ne*A′ P^810^-A **(D)** Trigger Loop from *M. jannaschii* **(E)** Trigger Loop from *N. equitans* (E) Fork Loop **3** from *M. jannaschii* (F) Fork Loop 3 from *N. equitans*.

The structural consequences of several of the substitutions were determined by comparing secondary structure propensities of sequences from *N. equitans* to equivalent domains from *M. jannaschii* (*mj*). Both species are hyperthermophiles thus containing similar sequence-encoded features that stabilize their protein structures at elevated temperatures. Markov Chain Monte Carlo (MCMC) simulations is the method of choice for a systematic and comprehensive exploration of conformational space (Boomsma et al., 2013) (Figure 3). As expected, the presence of a proline in the *ne*BH causes a substantial disruption of a region that displays high *a*-helical propensity in the *mj*BH (Tan et al., 2008). On a structural level, the presence of proline in *ne*A′ in position 810 (*ne*A′ P^810^) is predicted to cause a substantial destabilization of the *a*-helical conformation of the Bridge Helix in a slightly more N-terminal location (mostly affecting *ne*A′ R^808^; Figure 3B). Simulating a *ne* Bridge Helix with a “corrected” *in silico* point mutation (*ne*A′ P^810^-A) restores the predicted conformational population to one that is very close to the *mj* Bridge Helix (Figure 3C). This proves that the unusual conformational properties are predominantly due to *ne*A′ P^810^ position, rather than any of the other differences in the primary amino acid sequence. The region in the *ne* Bridge Helix most distorted corresponds to the orthologous region in *M. jannaschii* (*mj*A′-R^820^) which is a structure with one of the highest *a*-helical propensities of the entire domain (Figure 3A). High-throughput mutagenesis studies of *mj*A′-R^820^ in *mj*RNAP have shown it to be highly sensitive to point mutations, with only phenylalanine and tryptophane substitutions not resulting in substantial loss of catalytic activity (Tan et al., 2008; Weinzierl, 2013).

Similar comparisons of the *ne* and *mj* Trigger Loop conformations yield a less clear-cut result (Figure 3D,E), although the unusual position of a proline in the *ne* motif near the edges of the domain (*ne*A”-P^47^) again is likely to contribute a destabilizing influence (Figure 3E).

For *ne*FL3, the structural consequences of replacing highly conserved residues that are virtually invariant in other archaeal and eukaryotic polymerases in a non-conservative manner (for example, in FL3: C-D^445^, G-D^452^, V/I-R^458^, and N-A^460^; Figure 1C) suggest that this would cause distinct changes in the functional contributions of these residues to catalysis. Especially one of these substitutions *ne*B” G^452^-D is predicted to reduce the flexibility of the central region of *ne*FL3 considerably in comparison to the orthologous sequence of *mj*FL3 (Figures 3F,G). Similarly, Metal B contains two highly conserved acidic residues that coordinate of binding of the Mg^2+^ ion brought along by the incoming NTP, but in *N. equitans* one of them is converted to glutamine (*ne*A’ Q217) and thus is predicted bind the metal less strongly (Figure 1D).

Overall, based on previous insights from a range of structural and functional studies from archaeal and eukaryotic RNAPs representative of the majority of such organisms, a picture of a structurally diverged catalytic site in nanoarchaeal RNAPs emerges that suggests that the catalytic site may be more flexible in some areas (prolines in the *ne*Bridge Helix and *ne*Trigger Loop domains, stiffer in the diverged *ne*FL-3 domain and potentially compromised electrostatically by a diminished *ne*Metal-B motif).

### *In Vitro* Assembly of *ne*RNAP and High-Throughput “Sparse-Matrix” Sampling of *ne*RNAP Assay Conditions

**FIGURE 4:**
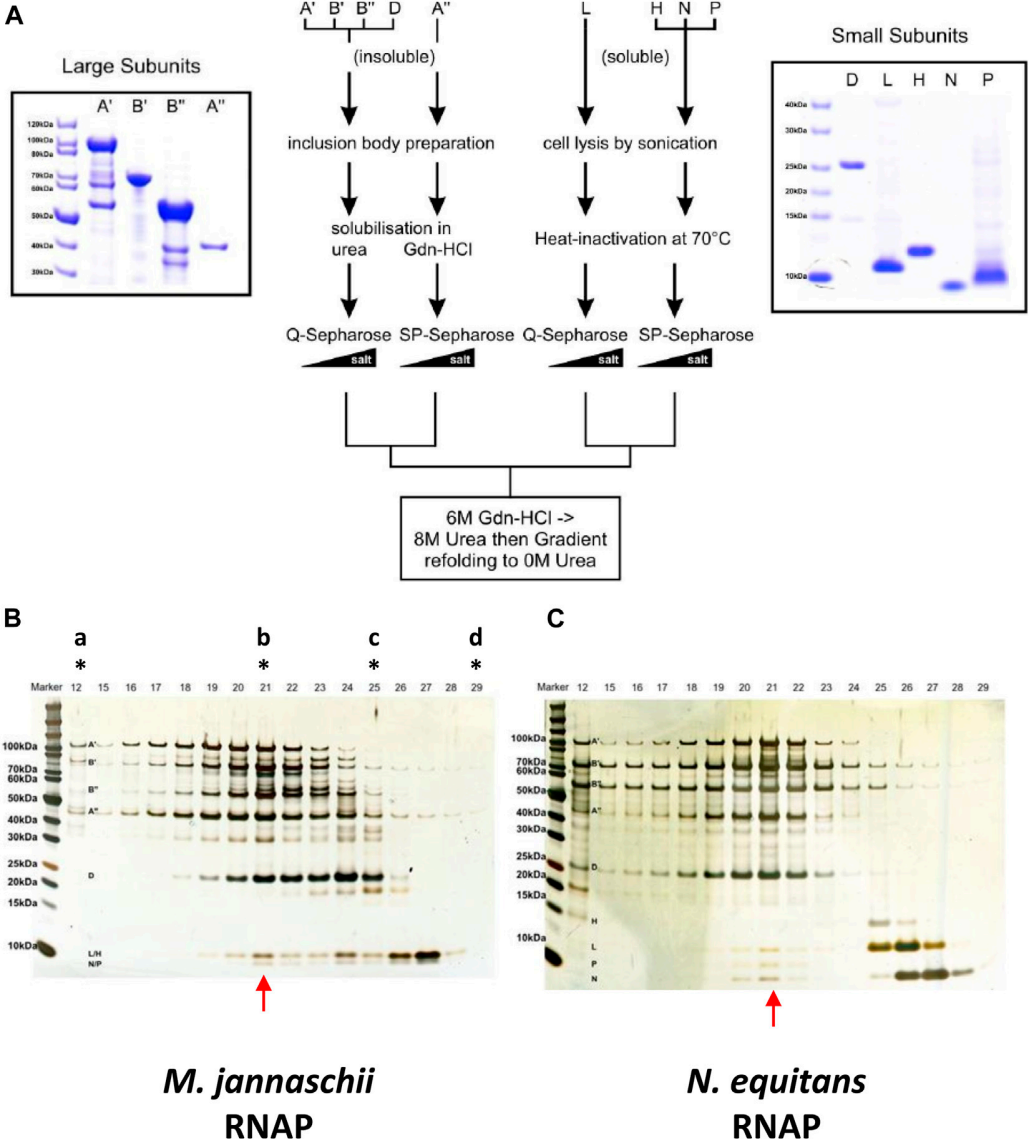
**(A)** Overview of the purification of *ne*RNAP subunits. The central scheme outlining the purification procedure for each subunit is flanked by Coomassie-stained gels of the purified subunits (left: *ne*A′, *ne*A”, *ne*B′ and *ne*B”; right: *ne*D, *ne*H, *ne*L, *ne*N and *ne*P) **(B)** Elution profile of the *mj*RNAP *in vitro* assembly reaction from a Superose-6 size-exclusion column (similar to (Werner and Weinzierl, 2002)) shown on a silver-stained Bis/Tris 4–12% gradient SDS-protein gel. Fraction 21 (indicated with red arrow) contains the fully assembled enzyme (and peak transcriptional activity; data not shown) as revealed by the presence of all subunits within a single fraction. The letters with stars on top show the fractions where the size exclusion markers (“a”, Blue Dextran 2,000 kDa; “b”, *ß*-amylase 200 kDa; “c”, carbonic anhydrase 25 kDa; “d”, cytochrome c 12.4 kDa) eluted **(C)** Similar to (B), but for the *ne*RNAP *in vitro* assembly.

The conformational distortions caused by potentially disruptive radical substitution suggest that the *Nanoarchaeum* RNAP may display only very low - or even no - catalytic activity. On the other hand, the presence of all known RNAP subunits in an otherwise minimal genome implies selective pressure responsible for maintaining an active transcriptional machinery. Technical problems with obtaining *N. equitans* in quantities sufficient for biochemical analysis preclude a direct purification of native enzymes from cells. We therefore decided to investigate this question by adopting the *in vitro* assembly approach that has been applied successfully for the assembly of RNAPs from other hyperthermophilic archaea (Werner and Weinzierl, 2002; Naji et al., 2007). The *in vitro* assembly of *ne*RNAP followed essentially the same procedure that we employed successfully in the past for *mj*RNAP (Werner and Weinzierl, 2002). Each of the subunits essential for catalytic activity was expressed as a recombinant protein in *E. coli*, followed by chromatographic purification and *in vitro* assembly by controlled dialysis from denaturing conditions (Figure 4A). Under these conditions, a portion of the *ne*RNAP subunits assembled into a complex that - comparable to *mj*RNAP (Figure 4B) - eluted as a distinct peak of activity during size exclusion chromatography (Figure 4C). As expected from its hyperthermophilic origin, the temperature optimum for catalytic activity was around 76°C (Supplementary Figures S3). Initial transcription experiments with *ne*RNAP suggested that the standard buffer conditions (120 mM potassium acetate, 10 mM magnesium acetate, 20 mM Tris-acetate, pH 8.6) were probably suboptimal because we observed a ∼ 7-fold lower specific activity for *ne*RNAP as compared to *mj*RNAP when assembled in parallel. We therefore attempted to optimize the assay conditions over a wider range of pH values, salt concentrations and in the presence of various additives. The concept of “sparse-matrix” sampling is well established in the macromolecular crystallization community where the method is used to identify the optimal (yet initially unknown) conditions to obtain macromolecular crystals for structural studies (Jancarik and Kim, 1991). Such approaches have also been employed usefully to identify optimal renaturation conditions (Hofmann et al., 1995), or for stabilizing macromolecular complexes (Chari et al., 2015). Here, we employed such a strategy to identify the best assay conditions for *ne*RNAP that included a wide range of different concentrations of various cations and anions, buffers at different pHs, and the presence of a variety of detergents and stabilizing reagents. A series of buffer sets (ICL-1, ICL-3, ICL-4, and ICL-5; Hampton Research), comprised of 386 different cocktails (see Supplementary Figures S2A–F for composition), were used as 10 x stock solutions after supplementing them with Mg^2+^ and Zn^2+^ in automated high-throughput transcription assays. Most of the mixtures include an inorganic or organic salt, a buffering compound (with pH ranges from 4.5 to 9.5) and a “precipitant”, such as polyethylene glycol. In our assays, the precipitant may display stabilizing effects on protein structure - especially quaternary structures - under hyperthermophilic assay conditions.

**FIGURE 5:**
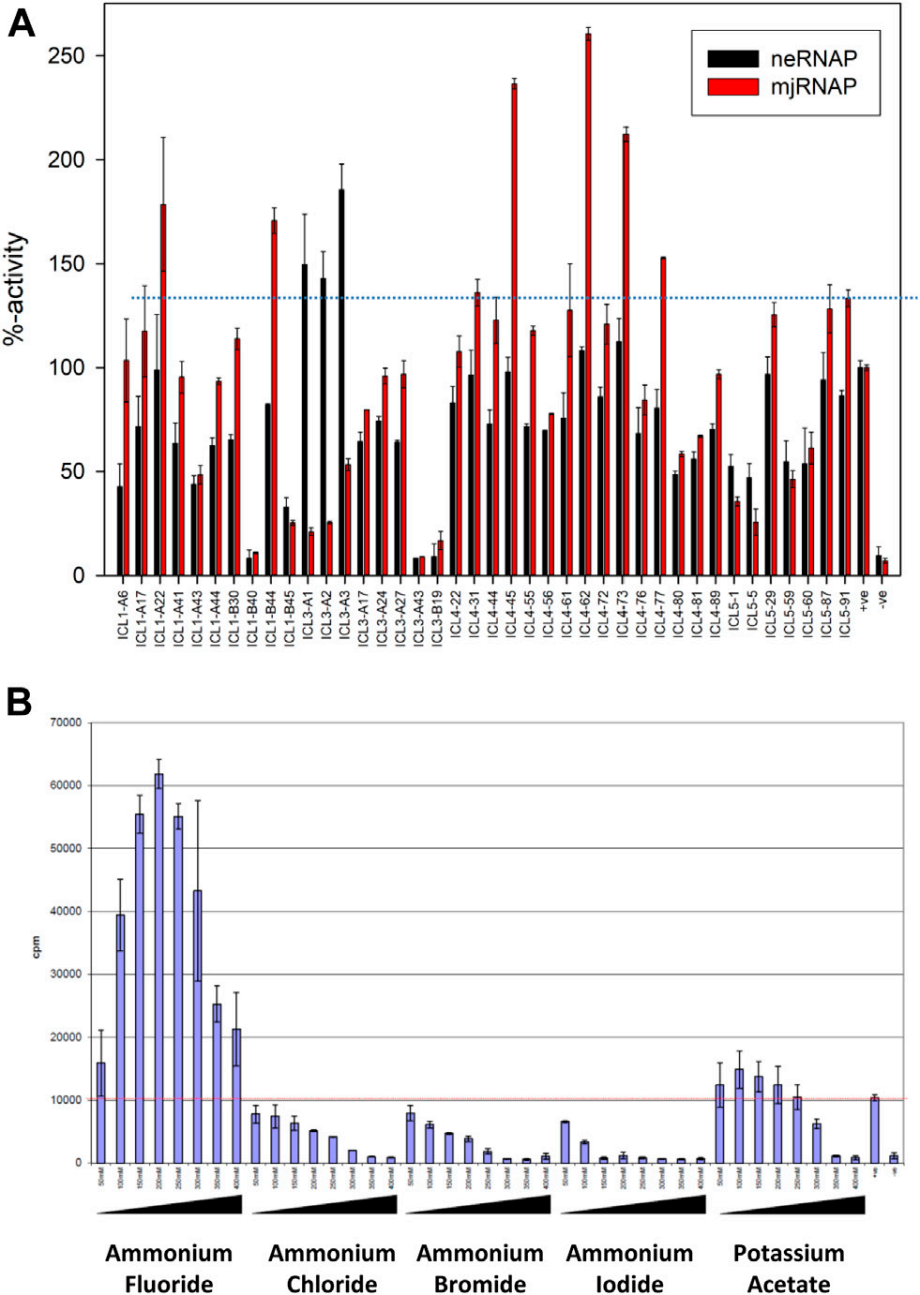
Effect of different assay buffer compositions on the catalytic activities of *ne*RNAP and *mj*RNAP **(A)** The transcriptional activity under “standard conditions” is defined as 100%. The activities of *ne*RNAP in this set of buffers is shown in black, and the performance of *mj*RNAP under the same conditions is shown in red (B) Fluoride-specific effect among halogen salts. The catalytic activity of *ne*RNAP at various salt concentrations ((50–400 mM) is shown, including ammonium fluoride, ammonium chloride, ammonium bromide, ammonium iodide and potassium acetate. Fluoride has the most distinct effect.

A summary of the results (see Supplementary Figure S4 for the complete data set) shows that *ne*RNAP had a clear preference for a group of three buffers (ICL-3 #A1, A2, A3) that contained 20 mM sodium fluoride, potassium fluoride and ammonium fluoride, respectively (Figure 5A). This apparent preference for fluoride is unique to *ne*RNAP because *mj*RNAP only performed moderately (30–50% in comparison to standard conditions) in these buffers (Figure 5A). To test this potential requirement for fluoride further, *ne*RNAP activity was assayed in transcription buffers containing varying amounts of fluoride salts. Optimal *ne*RNAP stimulation was achieved with 200–300 mM potassium fluoride or ammonium fluoride (Figure 5B and Supplementary Figure S5). The stimulation of *ne*RNAP activity by fluoride ions raised the question of whether other halogen ions (chloride, bromide, or iodide) would have a similar effect on *ne*RNAP. This, however, was not the case, suggesting that the stimulating effect on the catalytic activity of *ne*RNAP is indeed highly specific for fluoride.

## Discussion

Nanoarchaea are, in many ways, puzzling organisms. Their unique parasitic lifestyle has substantial effects on their cell- and genome size, which are both greatly minimized (Huber et al., 2002; Huber et al., 2003; Waters et al., 2003). Therefore, the cells depend on their host, *I. hospitalis* for many metabolites and precursors (Rawle et al., 2017). Analysis of the *N. equitans* genome has, however, revealed the presence of orthologs of all RNAP subunits and other components of the basal transcriptional machinery (TBP, TFB, and TFS; (Huber et al., 2002; Waters et al., 2003). It therefore looks as if *N. equitans* is capable of transcribing its own genome without help from its host cell in terms of imported basal transcription factors. Nevertheless, a number of key domains and motifs that constitute the active site of RNAP contains a distinct set of highly unusual and radical substitutions that appear to be deleterious to its catalytic activity.

**FIGURE 6:**
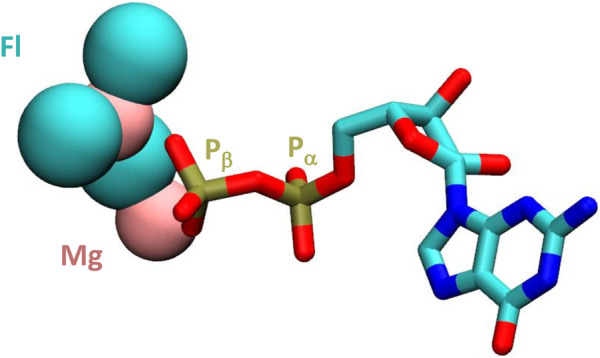
Structure of GDP complexed with magnesium and fluoride ions. The magnesium and fluoride ions are shown in pink and turqouize, respectively in van der Waals representation. The remainder of the GDP molecule is shown as a liquorice model. The *a* and *ß* positions of the phosphorus atoms are highlighted. Based on coordinates from PDB# 1OW3.

Here we show, by *in vitro* assembly of nanoarchaeal RNAP from recombinant subunits expressed in and purified individually from *E. coli*, that the resulting enzyme displays catalytic activity. The temperature, pH optimum and specific activity are within the expected range of a hyperthermophilic organism and comparable to a similar enzyme assembled from *M. jannaschii* (Werner and Weinzierl 2002). We therefore conclude that the changes in sequence, unusual as they may be, do not preclude catalytic activity. In a search for optimal assay conditions involving a sparse matrix approach, we discovered, however, an unexpected property: *ne*RNAP responded favourably to the presence of a high concentration of fluoride ions in the reaction buffer (optimal fluoride concentration for neRNAP ∼200–300 mM). Reports in the research literature from the 1970s describe a similar stimulatory effect of fluoride on adenylate cyclase (Drummond et al., 1971; Stalmans and Hers, 1975). These biochemical analyses showed that the reaction velocity (V_max_) of adenylate cyclase increased in the presence of fluoride but had no effect on the affinity (K_m_) for substrate molecules. It later became apparent that it was a regulatory subunit that was the target of the fluoride stimulation, and not adenylate cyclase itself (Hebdon et al., 1978; Sahyoun et al., 1981). The identity of the regulatory protein turned out to be a subunit of a membrane bound, heterotrimeric G-protein complex. This G-protein is a gtpase and upon binding of GTP activates adenylate cyclase activity. The stimulatory effect of fluoride is believed to be the result of the ability of fluoride to form multi-fluorinated complexes with metal ions, such as Mg^2+^ (Antonny et al., 1993). Such “MgFx” complexes are capable of mimicking the *γ*-phosphate of a GTP molecule (Higashijima et al., 1991) and are thus able to occupy the phosphate binding pocket of the nucleotide-binding site of the G-protein. Several other G-protein dependent regulatory enzymes (such as Erk, Rho, Ras) have been shown to respond to fluoride in such a way (Bogatcheva et al., 2006). Fluoride has also been shown to bind to pyrophosphate (Baykov et al., 2000). We therefore hypothesize that the stimulation of transcription by high levels of fluoride ions may have a comparable cause in nanoarchaal RNAP. The presence of mono- or multi-fluorinated NTP complexes (see Figure 6 for a GDP-based example) may assist with binding of NTPs to a structurally more flexible active site in *ne*RNAP and/or help to stabilize some transition complexes in the nucleotide addition cycle. It is possible that especially the binding of Mg^2+^ ions to the divergent Metal B motif could be influenced in such a manner. According to such a model, the observed lack of effect of fluoride on the catalytic activity *mj*RNAP would reflect the fact that “conventional” RNAPs do not require this kind of assistance for their catalytic sites to operate.

Future studies will focus on the potential interplay between fluoride, Mg^2+^ and NTPs, as well as defining in more detail which of the diverged motifs is most susceptible to this effect. By replacing some of the substitutions - either individually or in groups - with residues that are normally found in their position in other RNAPs, we will be able to study which of them are most likely to be responsible for this unusual behavior of *ne*RNAP in presence of fluoride.

## Data Availability

The datasets presented in this study can be found in online repositories. The names of the repository/repositories and accession number(s) can be found in the article/Supplementary Material.
