# Supplementary material for: Transcribing Genes the Hard Way: In Vitro Reconstitution of Nanoarchaeal RNA Polymerase Reveals Unusual Active Site Properties
Source: Front Mol Biosci. 2021 May 11;8:669314. doi: 10.3389/fmolb.2021.669314 (PMC8204694; doi:10.3389/fmolb.2021.669314)
Supplement: Supplementary file 1 [file Presentation1.PPTX]

## Slide 1
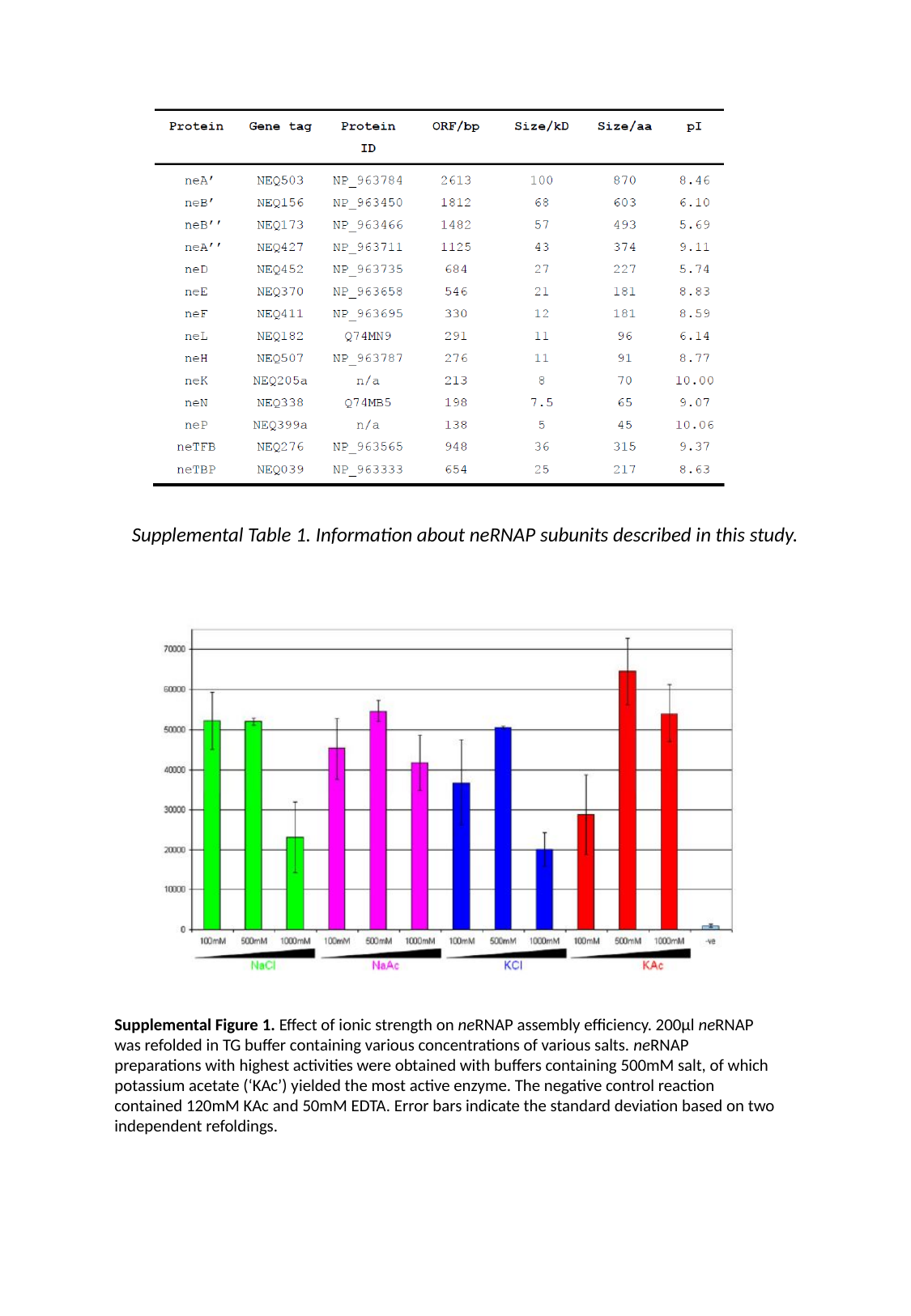

Supplemental Table 1. Information about neRNAP subunits described in this study.
Supplemental Figure 1. Effect of ionic strength on neRNAP assembly efficiency. 200μl neRNAP was refolded in TG buffer containing various concentrations of various salts. neRNAP preparations with highest activities were obtained with buffers containing 500mM salt, of which potassium acetate (‘KAc’) yielded the most active enzyme. The negative control reaction contained 120mM KAc and 50mM EDTA. Error bars indicate the standard deviation based on two independent refoldings.

## Slide 2
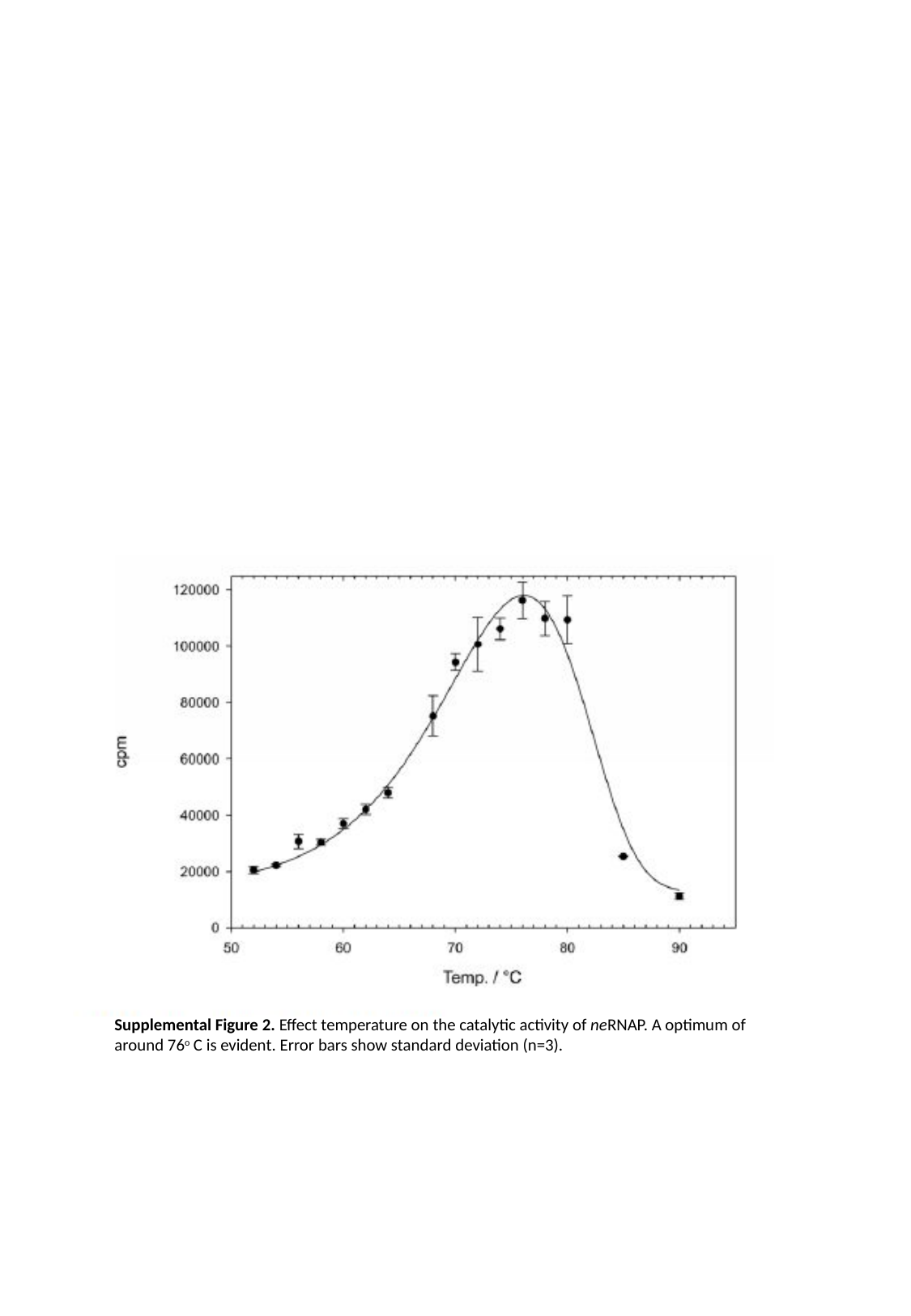

Supplemental Figure 2. Effect temperature on the catalytic activity of neRNAP. A optimum of around 76o C is evident. Error bars show standard deviation (n=3).

## Slide 3
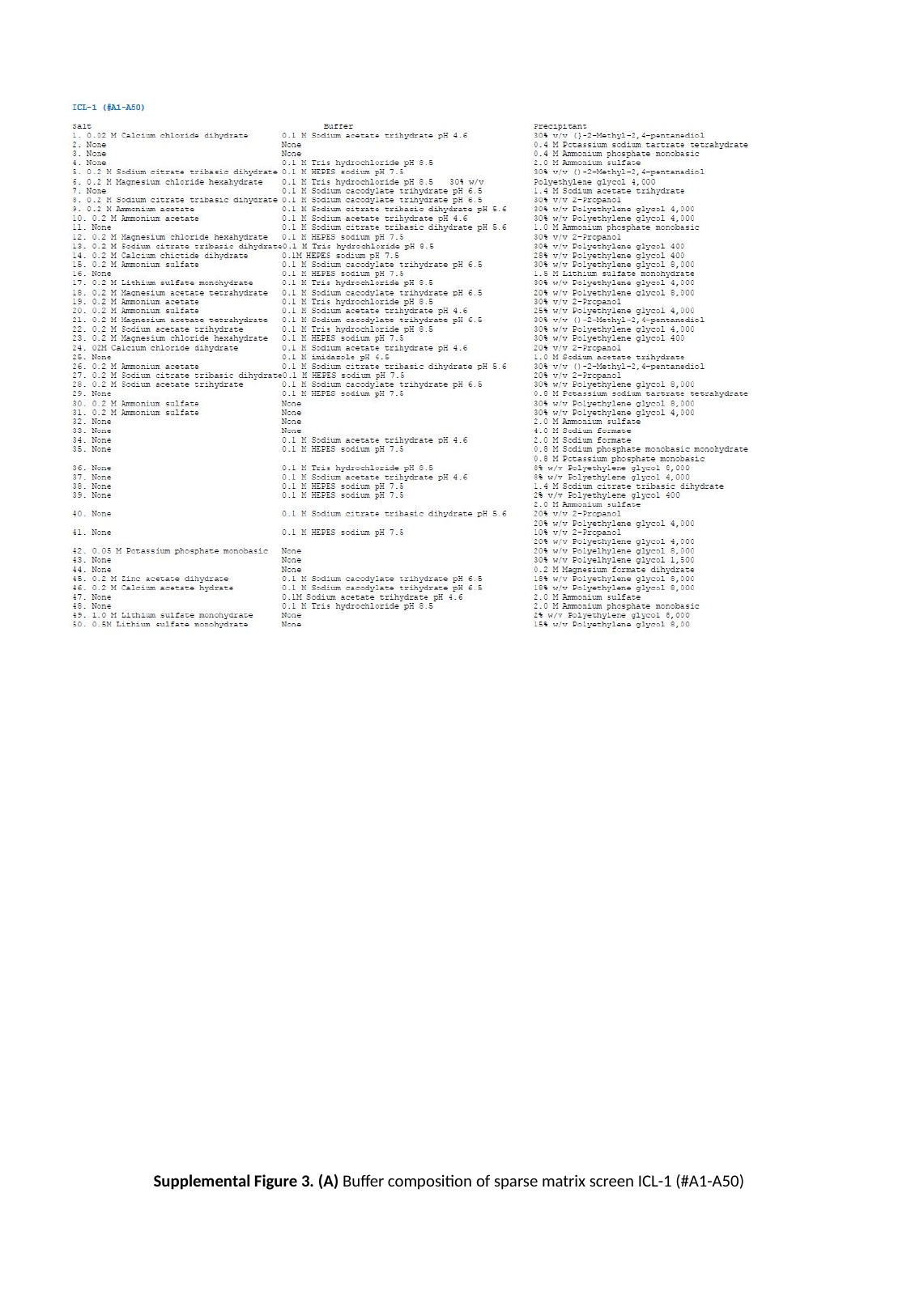

Supplemental Figure 3. (A) Buffer composition of sparse matrix screen ICL-1 (#A1-A50)

## Slide 4
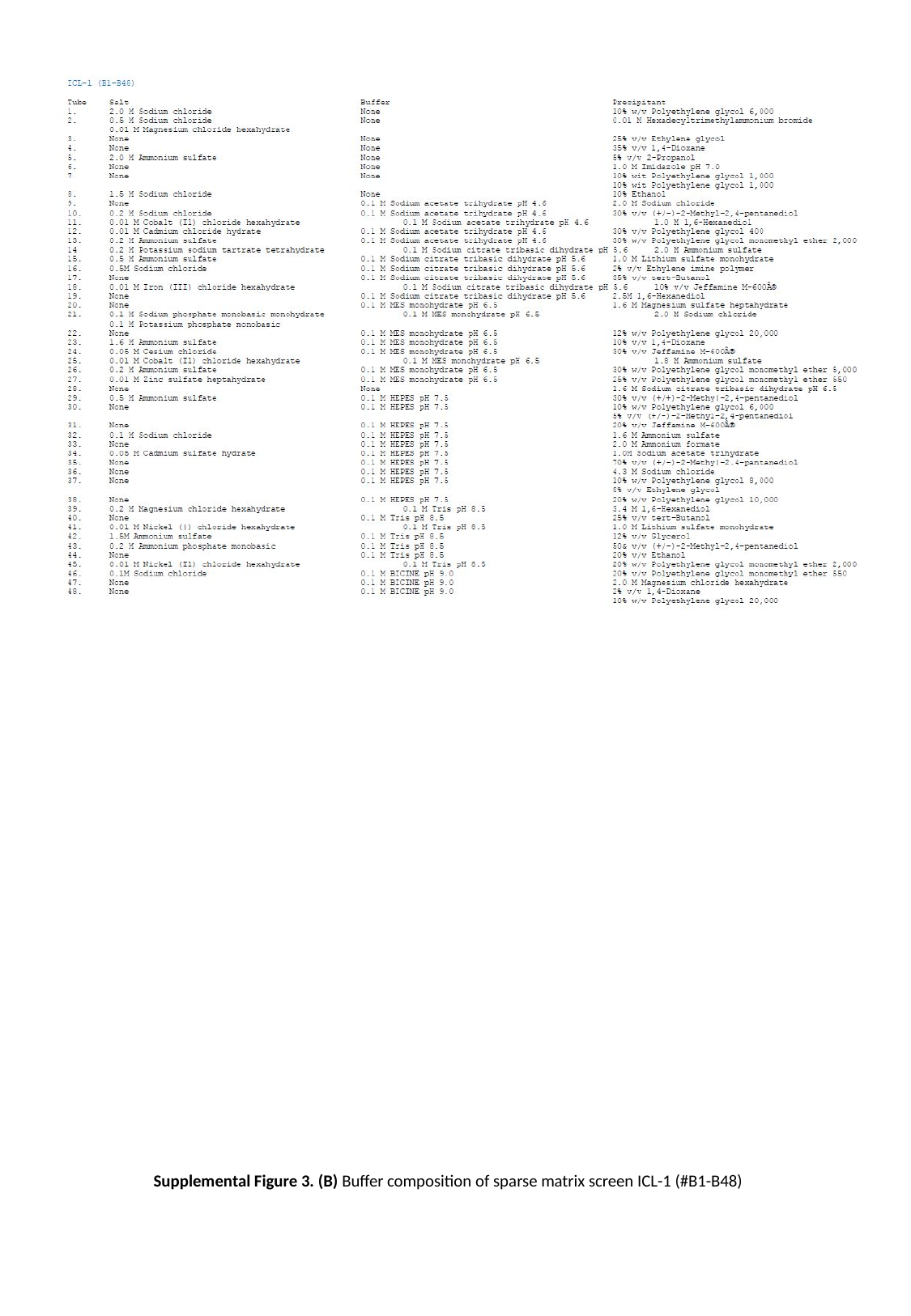

Supplemental Figure 3. (B) Buffer composition of sparse matrix screen ICL-1 (#B1-B48)

## Slide 5
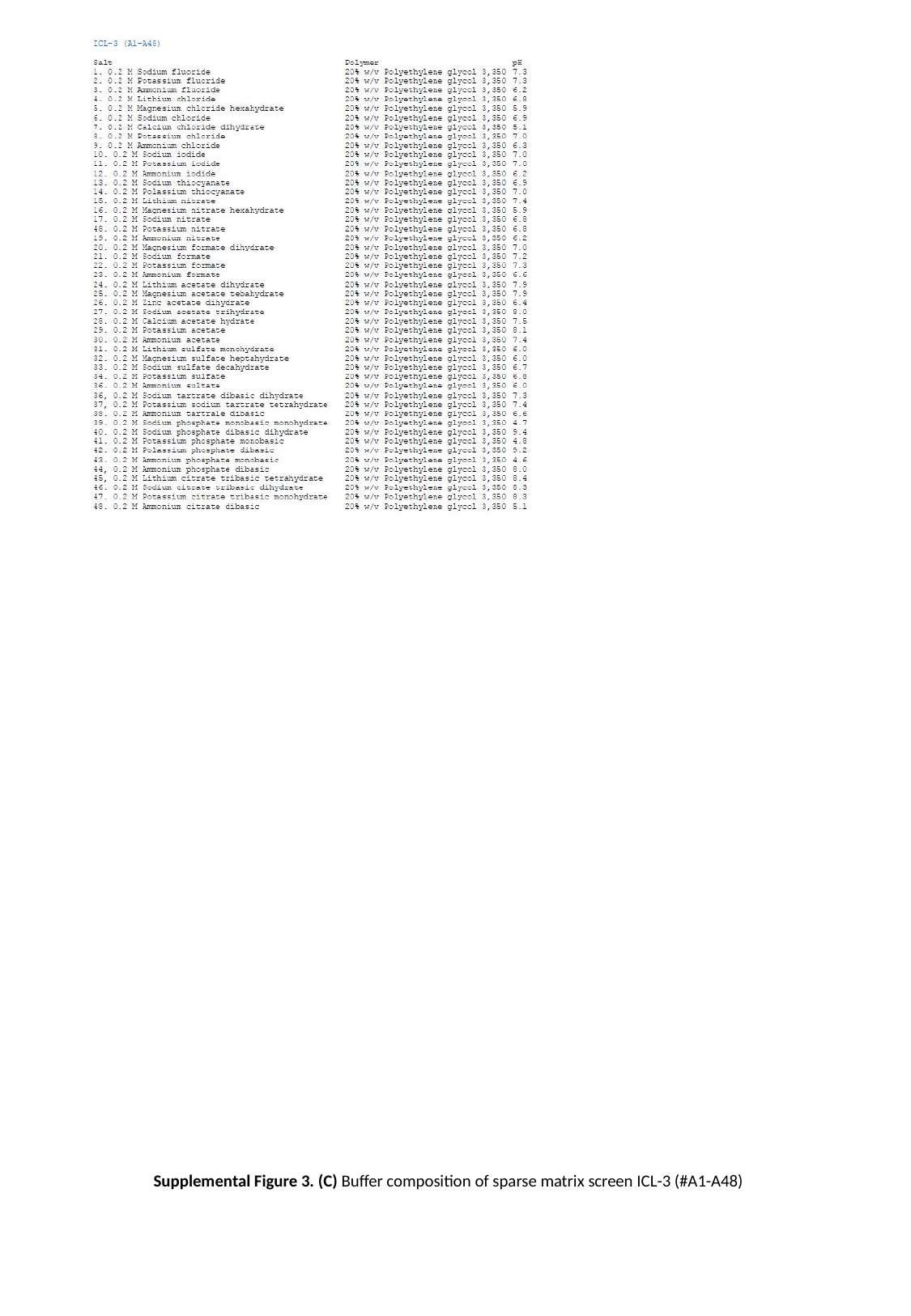

Supplemental Figure 3. (C) Buffer composition of sparse matrix screen ICL-3 (#A1-A48)

## Slide 6
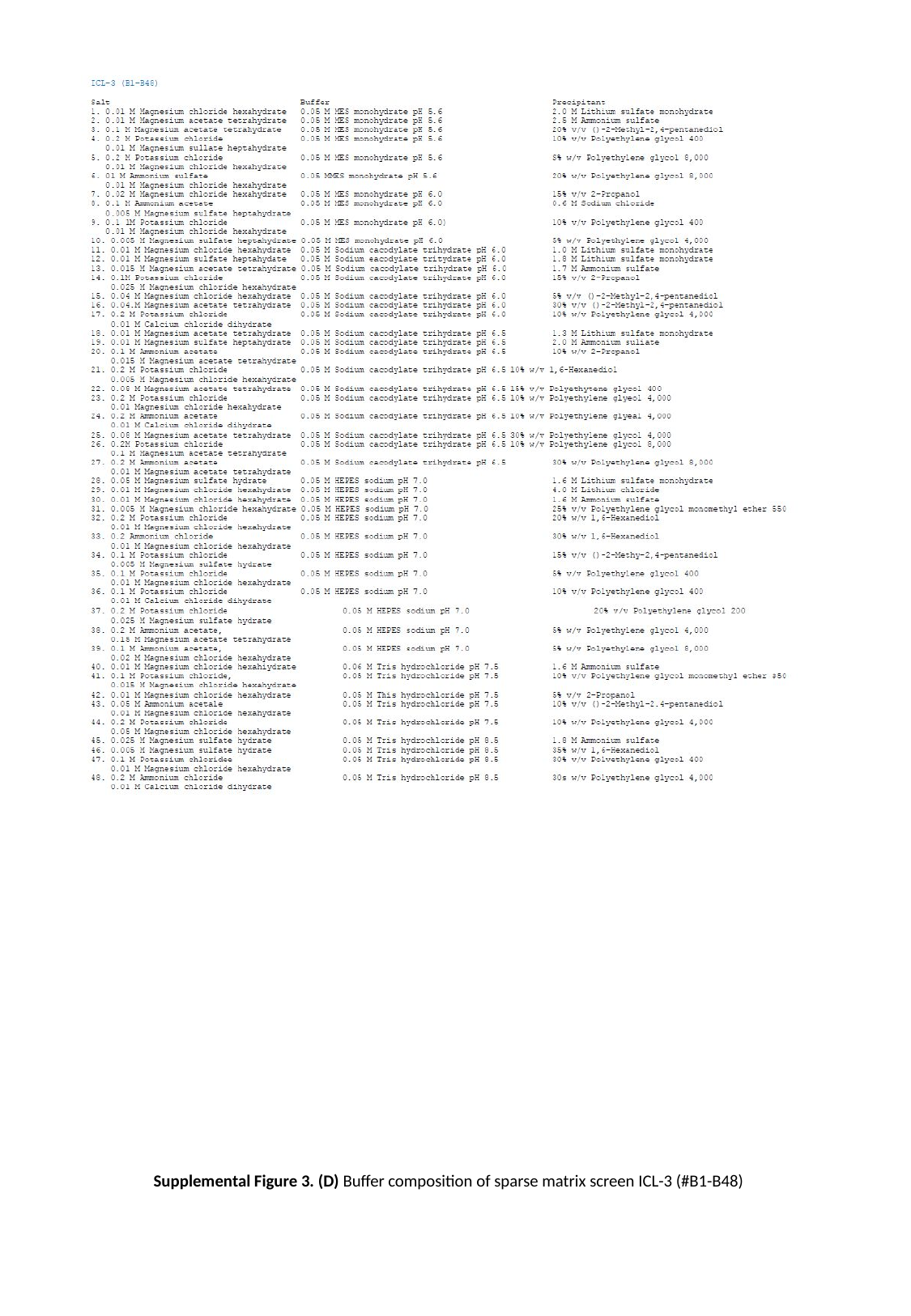

Supplemental Figure 3. (D) Buffer composition of sparse matrix screen ICL-3 (#B1-B48)

## Slide 7
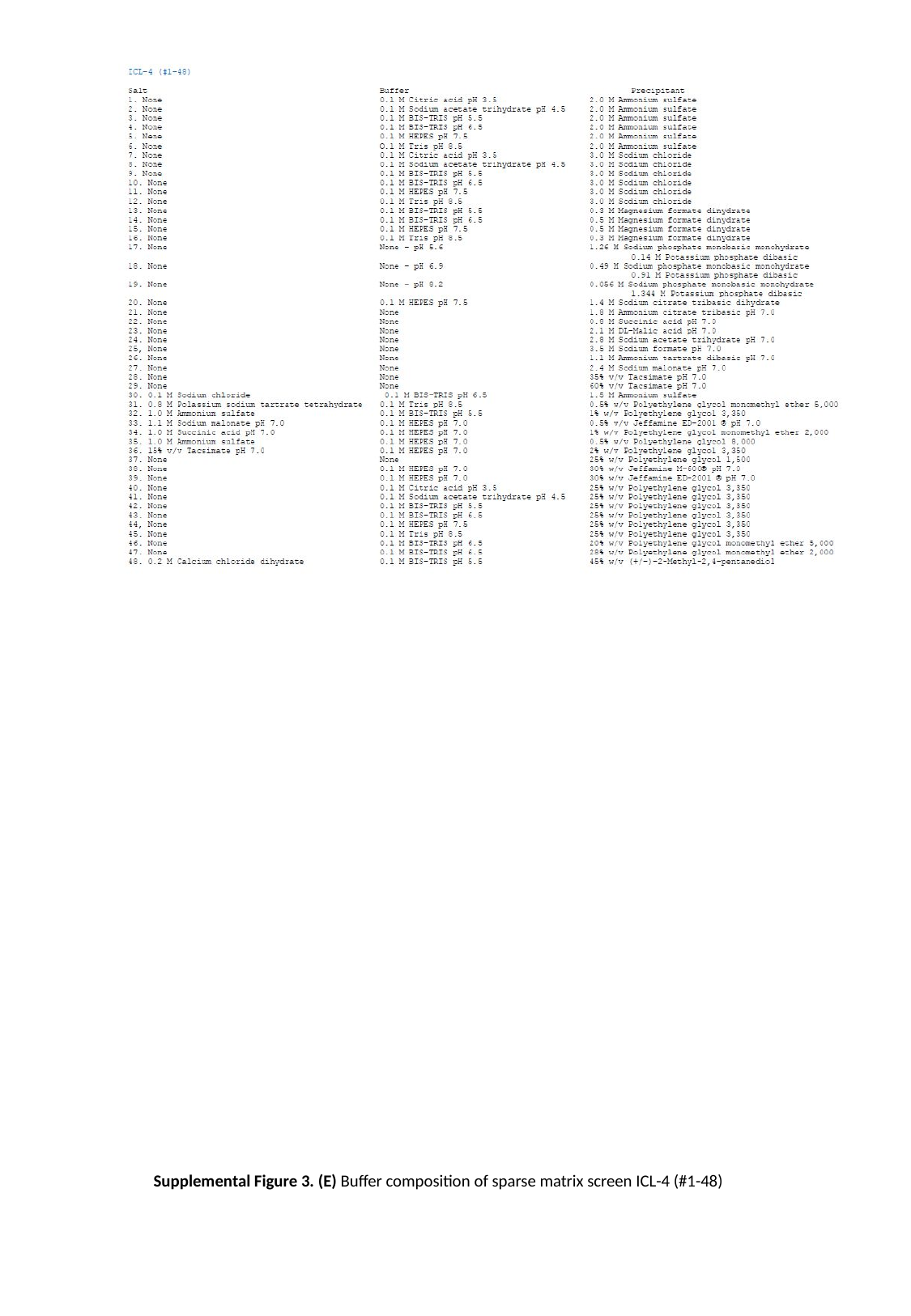

Supplemental Figure 3. (E) Buffer composition of sparse matrix screen ICL-4 (#1-48)

## Slide 8
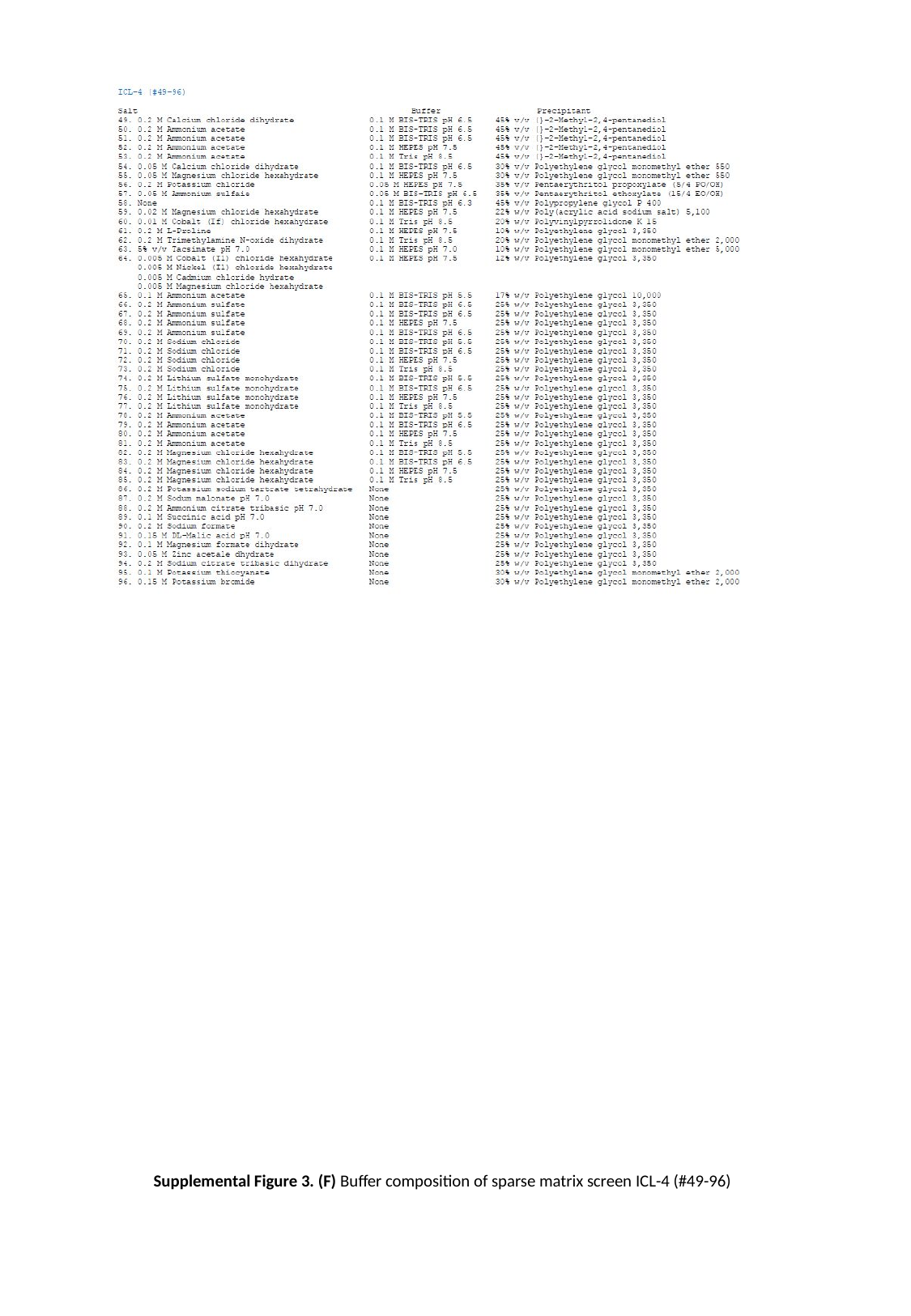

Supplemental Figure 3. (F) Buffer composition of sparse matrix screen ICL-4 (#49-96)

## Slide 9
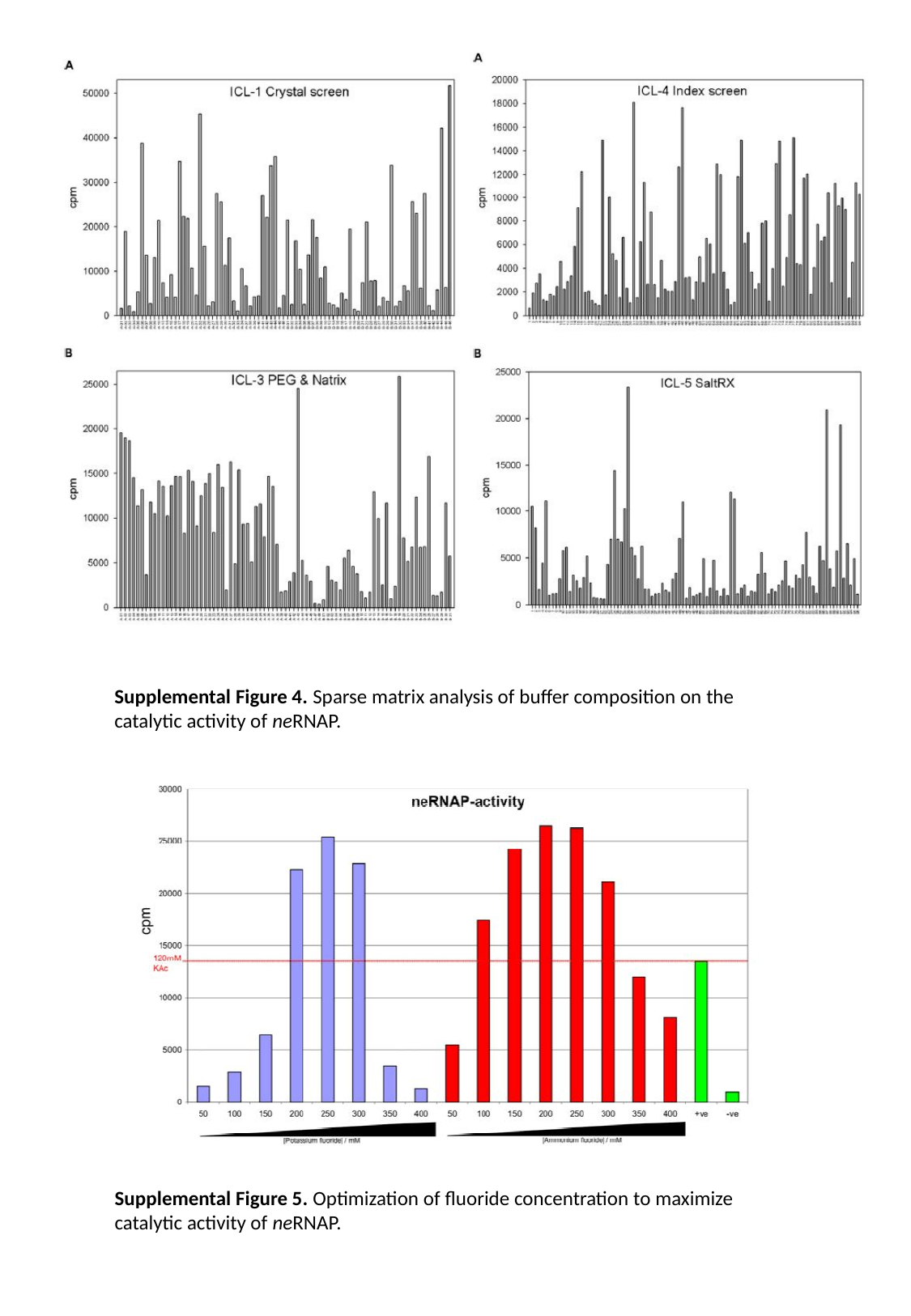

Supplemental Figure 4. Sparse matrix analysis of buffer composition on the catalytic activity of neRNAP.
Supplemental Figure 5. Optimization of fluoride concentration to maximize catalytic activity of neRNAP.
